# Scientific and technological advances in the development of sustainable disease management tools: a case study on kiwifruit bacterial canker

**DOI:** 10.3389/fpls.2023.1306420

**Published:** 2024-01-11

**Authors:** Miguel G. Santos, Marta Nunes da Silva, Marta W. Vasconcelos, Susana M. P. Carvalho

**Affiliations:** ^1^ GreenUPorto—Sustainable Agrifood Production Research Centre/Inov4Agro, DGAOT, Faculty of Sciences of the University of Porto, Vairão, Portugal; ^2^ Universidade Católica Portuguesa, CBQF – Centro de Biotecnologia e Química Fina – Laboratório Associado, Escola Superior de Biotecnologia, Porto, Portugal

**Keywords:** *Actinidia* spp., antimicrobial molecules, microbial biological control agents, plant breeding, plant elicitors, precision agriculture, *Pseudomonas syringae* pv. *actinidiae*, sustainable horticulture

## Abstract

Plant disease outbreaks are increasing in a world facing climate change and globalized markets, representing a serious threat to food security. Kiwifruit Bacterial Canker (KBC), caused by the bacterium *Pseudomonas syringae* pv. *actinidiae* (Psa), was selected as a case study for being an example of a pandemic disease that severely impacted crop production, leading to huge economic losses, and for the effort that has been made to control this disease. This review provides an in-depth and critical analysis on the scientific progress made for developing alternative tools for sustainable KBC management. Their status in terms of technological maturity is discussed and a set of opportunities and threats are also presented. The gradual replacement of susceptible kiwifruit cultivars, with more tolerant ones, significantly reduced KBC incidence and was a major milestone for Psa containment – which highlights the importance of plant breeding. Nonetheless, this is a very laborious process. Moreover, the potential threat of Psa evolving to more virulent biovars, or resistant lineages to existing control methods, strengthens the need of keep on exploring effective and more environmentally friendly tools for KBC management. Currently, plant elicitors and beneficial fungi and bacteria are already being used in the field with some degree of success. Precision agriculture technologies, for improving early disease detection and preventing pathogen dispersal, are also being developed and optimized. These include hyperspectral technologies and forecast models for Psa risk assessment, with the latter being slightly more advanced in terms of technological maturity. Additionally, plant protection products based on innovative formulations with molecules with antibacterial activity against Psa (*e.g*., essential oils, phages and antimicrobial peptides) have been validated primarily in laboratory trials and with few compounds already reaching field application. The lessons learned with this pandemic disease, and the acquired scientific and technological knowledge, can be of importance for sustainably managing other plant diseases and handling future pandemic outbreaks.

## 1 Introduction

Climate change and the global food trade are exacerbating the impact of plant diseases worldwide, facilitating the dissemination and increasing the unpredictability of the occurrence of many phytopathogenic microorganisms, which constitute a serious threat to global food security (Anderson et al., 2020). Numerous plant diseases still lack sustainable management strategies and rely on the frequent use of hazardous chemical pesticides. These pesticides are often poorly effective and are persistent in the environment, where they affect the biota and degrade the quality of water masses and soils (Urionabarrenetxea et al., 2022). Moreover, plant diseases also lead to high mitigation costs due to control measures and have downstream impacts on human health (Ristaino et al., 2021).

Among the causal agents of plant diseases, bacteria have been subject to particular concern due to the emergence of lineages which have been severely affecting several agroecosystems in recent years. The Kiwifruit Bacterial Canker (KBC) constitutes a representative example for being the most devastating disease currently impairing kiwifruit (*Actinidia* spp.) cultivation worldwide (Donati et al., 2020), after the emergence of a highly virulent biovar of the bacterium *Pseudomonas syringae* pv. *actinidiae* (Psa). Psa is a Gram-negative, rod-shaped and flagellated bacterium, which utilizes motility and biofilm formation for epiphytic fitness and survival (Sawada and Fujikawa, 2019; Krishna et al., 2022). After this epiphytic stage, the pathogen infects the host through natural openings such as stomata, lenticels, hydathodes, and plant-damaged tissues (*e.g*., broken trichomes, pruning wounds), for later colonizing both xylem and phloem (Gao et al., 2016; Cellini et al., 2021). In 2008, there was a very serious outbreak in Italy (caused by Psa biovar 3), and KBC became a pandemic disease, leading to severe economic losses in all main kiwifruit-producing countries, such as China, New Zealand, Italy, Chile, France, Portugal, Spain and South Korea (Abelleira et al., 2015; Cunty et al., 2015; McCann et al., 2017; Vanneste, 2017; Kim et al., 2018). Soon after the outbreak, the implementation of strict phytosanitary orchard management routines, conjugated with copper treatments and application of antibiotics (where allowed), was very important in containing Psa dispersion. Nonetheless, since 2012, owed to the increasing bacterial resistance to those chemicals, and the Psa ability to disseminate and infect kiwifruit plants, this pathogen is included up to now in the A2 List from the European and Mediterranean Plant Protection Organization (EPPO), which identifies the organisms recommended for regulation as quarantine pests and diseases (EPPO, 2012).

In the last two decades the scientific community and commercial sector have made a good progress in developing and bringing to the market more environmentally friendly alternatives to the use of copper and antibiotics. The gradual substitution of susceptible *Actinidia* cultivars with Psa-tolerant ones (plant breeding programs) (Wang et al., 2020), and the application of plant elicitors for triggering plant defenses against the Psa (Beatrice et al., 2017), are supporting a slow recovery of the kiwifruit sector (Nunes da Silva et al., 2022b). More recently, the development of eco-friendly KBC control methods has been focused on screening and testing a myriad of emerging molecules, compounds, and Microbial Biological Control Agents (MBCAs) with potential to reduce *in planta* Psa load, motility and spread capacity, resulting from their bacteriostatic, bactericidal and competition activity. These include plant Essential Oils (EOs) and other bioactive compounds, phages and Antimicrobial Peptides (AMPs) (Monchiero et al., 2015; Lovato et al., 2019; Pereira et al., 2021). In addition, state-of-the-art gene-editing techniques, currently giving the first steps in *Actinidia* (Michelotti et al., 2022), hold promise to constitute powerful tools for accelerating kiwifruit breeding programs, despite the European strict regulatory constraints (Callaway, 2018). Finally, the development of forecast models and early disease detection methods have the potential for integrating control strategies, allowing anticipated action to contain Psa spread at plant, orchard and regional levels (Do et al., 2016; Narouei-Khandan et al., 2022; Reis-Pereira et al., 2022). For effective mitigation of KBC, these methods may be combined in an orchestrated manner to enhance plant defense capacity and to suppress Psa virulence.

In this literature review we provide an in-depth and critical analysis on the scientific and technological progress made, and the current limitations, regarding the tools being explored for a sustainable disease management – focusing on KBC as a case study. A set of opportunities and threats are also presented, which may provide guidance for researchers, policy makers and regulators on the path ahead towards the mitigation of future plant disease outbreaks.

## 2 State-of-the-art on sustainable disease management tools to mitigate KBC

An effective phytosanitary program requires strategies for avoiding pathogen spread, for developing pathogen-tolerant cultivars and the release of commercial products which are biologically safe to humans and to the environment. The containment of virulent pathogens such as Psa demands the implementation of measures to be adopted at local, regional, national and international levels. Moreover, other agronomical aspects related to crop maintenance in the field need also to be addressed. In *Actinidia*-Psa, for example, favoring less dense canopies and avoiding excessive plant vigor have been pointed out as important aspects to consider in places with higher pathogen pressure (Mauri et al., 2016). In addition, optimized nutritional regimens should also be developed aiming at increasing *Actinidia* tolerance to Psa (Mauri et al., 2016; Gu et al., 2021; Nunes da Silva et al., 2021b). These aspects have been addressed in recent reviews (Ristaino et al., 2021; Nunes da Silva et al., 2022b). Complementary to this, in the present review we focused on precision agriculture and biotechnological tools, namely: (i) the application of early detection tools, (ii) modelling for predicting the distribution and potential spread of Psa globally, (iii) the advances and prospects about plant breeding for Psa tolerance, and (iv) numerous molecules and MBCAs being tested for enhancing plant defense or suppressing the Psa pathogenicity.

### 2.1 Precision agriculture

#### 2.1.1 Early disease detection tools

In the last decade, many studies and commercial applications arose on the use of non-invasive methodologies for early disease detection (Mahlein et al., 2018; Reis-Pereira et al., 2021). These methodologies allow to generally assess plant fitness as they detect plant physiological, nutritional and structural alterations, based on spectral characteristics (reflectance, transmittance and absorbance) of the plant tissues (Bergsträsser et al., 2015). The optical sensing systems being currently applied explore different technologies including: (i) techniques that provide images, namely Red-Green-Blue (RGB) and thermal cameras, multispectral (lower resolution) cameras, hyperspectral (higher resolution) cameras and fluorescence imaging devices; (ii) non-imaging techniques, based on visible (VIS) and infrared (IR) spectroscopy and fluorescence spectroscopy (Gogoi et al., 2018). Current technological sophistication allows to measure data within a spectral range from 325 to 2500 nm of wavelength of electromagnetic radiation, with very high resolution (Mahlein, 2016; Reis-Pereira et al., 2022). The rationale behind the application of these technologies lies in modifications on the composition or structure of plant tissues resulting from the action of stressors (either of biological or non-biological origin). In the best possible scenario, the analyses of the optical properties of plants would enable to detect early plant infection processes, before symptoms could be detected by the human vision, thus allowing anticipated phytosanitary measures to be applied in the field.

Satellite imagery has been used for monitoring Psa incidence in kiwifruit orchards, and a series of vegetation indices that could be related to disease development were identified (Taylor et al., 2014). Multi-spectral imagery, using wavebands ranging from VIS to near-infrared region (NIR) of the electromagnetic spectrum, were analyzed and compared with shapefiles, resulting from precise mapping of Psa-positive samples collected along orchards in New Zealand. The authors achieved reasonable detection accuracies, ranging from 75% to 79%, by using binomial logistic regression in the modelling of the vegetation indices. However, the method was more limited during stages when vine vigor was more stationary (Taylor et al., 2014).

Thermal imagery (or IR thermography) can be used to assess leaf tissue temperature, which is correlated to evapotranspiration (Mahlein, 2016). Therefore, slight modifications in transpiration caused by Psa infection can be detected with IR cameras before symptoms can be visualized in the field. For instance, Maes et al. (2014) found that Psa could be detected at the leaf level by thermal imaging, as early as two days after exposure, through the identification of cold spots on the leaf surface not showing visual symptoms. At the orchard level, this technology was also validated in the mapping of affected zones with Psa-infected areas showing colder canopies (from 1 °C to 3 °C, approximately), and a tendency for higher Psa incidence in outer parts of the canopies was also observed (Maes et al., 2014). Nonetheless, this approach cannot discriminate Psa infection from other stresses that induce changes in evapotranspiration (*e.g.*, heat or drought stress).

Given that numerous factors can cause physiological and anatomical disturbances, several authors have dedicated their work to try to identifying spectral signatures which ultimately would assist the disease diagnosis (Mahlein et al., 2013; Ashourloo et al., 2014; Al-Saddik et al., 2017). Recently, this approach was also applied for early detection of Psa infection in a two-week monitoring in kiwifruit orchards in Portugal (Reis-Pereira et al., 2022). For this, the authors utilized a portable spectroradiometer (non-imaging approach) for measuring the spectral reflectance of *A. chinensis* var. *deliciosa* leaves within a spectral range between 325-1075 nm, which includes part of the Ultraviolet (UV) region, the entire VIS and part of the NIR region of the electromagnetic spectrum. By applying several mathematical and computational tools, the authors were able to differentiate symptomatic from asymptomatic vines, with an accuracy of 85%. The most discriminatory regions of the electromagnetic spectrum were in the blue (350–500 nm), green (500–600 nm), red (600–750 nm) and NIR (>750 nm) regions. These alterations might be related to diminished content of photosynthetic pigments, cell death and other structural damages (Wang et al., 2018b; Reis-Pereira et al., 2021). It was recently shown that the reduction in carotenoids of kiwifruit leaves resulting from Psa infection could be detected recurring to a special spectroscopic technique called Surface-Enhanced Raman Spectroscopy (SERS), only three days post Psa inoculation, when leaves were still asymptomatic (Jiang et al., 2023).

Nevertheless, care is advised when taking conclusions from experiments conducted under field conditions, where a variety of biotic and abiotic stresses may interfere with spectral signatures. Studies utilizing plant inoculations under controlled conditions are needed for more precisely elucidating the alterations of the optical properties of plant tissues during the first stages of infection, providing a basis of knowledge for improving field surveillance. Also, the development of robust discriminative spectral wavebands capable of differentiating Psa infection from other plant-pathogens interactions is still required (Al-Saddik et al., 2019). In this respect, the use of emerging techniques of deep machine learning (convolutional neuronal networks) may greatly improve the application of early disease detection techniques to mitigate KBC (Mohanty et al., 2016; Boulent et al., 2019; Toda and Okura, 2019).

#### 2.1.2 Forecast models for Psa risk assessment

Forecast modelling has long been used to predict the spread and incidence of several plant pathogens, allowing to better plan and anticipate the set of necessary measures for a more effective phytosanitary control. Concerning Psa management, the ‘OsiriS’ (Marcon et al., 2015) and ‘OttaviO’ (Cacioppo et al., 2015) sensing systems were successfully tested by Cacioppo et al. (2015) in a field trial. The systems measured pedoclimatic parameters (including air temperature, relative humidity, leaf wetness, wind speed, photosynthetically active radiation, soil tension, pH, and electrical conductivity), which can be remotely accessed for decision-making. This integrated approach was used to predict Psa infection and to decide when to apply copper treatments. The authors claimed that it resulted in a 60% decrease in Psa symptoms and in the reduction of costs associated with copper application.

Another weather-based model for predicting the risk of Psa infection (Psa-V risk model) was developed and further tested in New Zealand, Italy and Korea (Beresford et al., 2017; Kim et al., 2018; Antoniacci et al., 2019). This model conjugates the concept of multiplication capacity (M index, which estimates pathogen population growth based on accumulated air temperature, leaf surface wetness and relative air humidity) and Psa dispersal. The risk of infection (R index) depends on the total rainfall of the last 24 h, which should be above 1 mm, otherwise the R index would be considered null (Beresford et al., 2017). This model was further optimized to avoid false positives during summer due to high temperatures that, not being favorable to Psa growth, were not taken into consideration in the risk estimation. Accumulation of rain had also to be re-considered, not only because of inoculum reductions during heavy rainfalls but also due to the influence of temperature on humidity persistence on the plant surface (Kim et al., 2018).

Modelling methods such as CLIMEX, MaxEnt and multimodel framework (MMF) were also used to predict the global spread and distribution of Psa (Narouei-Khandan et al., 2013; Narouei-Khandan et al., 2022). Narouei-Khandan et al. (2013) utilized CLIMEX to generate ecoclimatic indices to predict the favorableness of a specific region to be colonized by Psa, and MaxEnt to predict Psa distribution using a set of selected bioclimatic variables combined with geo-referencing. However, discrepancies between both models were observed for regions with different climatic conditions than those in which the models were originally trained (Narouei-Khandan et al., 2013), reinforcing the importance of calibration and revalidation in forecast modelling. Such discrepancies between modelling methods motivated a different approach where CLIMEX, MaxEnt and MMF were utilized for developing a consensus model (Narouei-Khandan et al., 2022). This model relied on prediction agreement among different methods to “dilute” the fragilities from each individual modelling tool, resulting in higher sensitivity and fewer false negatives through this approach (Narouei-Khandan et al., 2022).

Regarding modelling for Psa infection and spread, considerations must be taken on factors that could exacerbate disease development, such as frost, wind, or genotypic susceptibility (Woodcock, 2016). Freezing temperatures and their duration were considered in the model from Do et al. (2016) to predict damage accumulation in Psa-infected canes from *A. chinensis* var. *deliciosa* ‘Hayward’ and *A. chinensis* var. *chinensis* ‘Hort16A’. However, this model was only accurate in predicting accumulated damage (lesion extension and rate of necrosis) in the less susceptible cultivar (‘Hayward’) but not in the most susceptible one (‘Hort16A’) (Do et al., 2016).

One of the biggest challenges in forecast modelling is the adjustment to new contexts for reliable extrapolation, further aggravated under scenarios of climate change. Considering future climatic scenarios, MaxEnt was used in combination with ArcGIS^®^ (ESRI, 2014) for modelling Psa distribution in China, resulting in mapping regions according to four categories of suitability to harbor the pathogen (Wang et al., 2018a). More recently, Qin et al. (2020) presented their projections of Psa distribution in China by 2050, using an ensemble model resulting from three modelling algorithms combining a set of bioclimatic variables. The authors highlighted several aspects – such as precipitation from late winter to early spring, and average temperature during the warmest quarter – further indicating that environmental variables contribute to a greater extent to model accuracy when translated into bioclimatic features with more ecological meaning (Qin et al., 2020). These projections should be extended to other regions for a better action plan against Psa at both regional and global levels.

### 2.2 Plant breeding

Plant breeding is a cornerstone of an integrated approach to mitigate agronomical pathogens, including Psa. Whereas previous breeding programs were mainly oriented towards improving fruit organoleptic properties, after the complete collapse of the most sensitive cultivar worldwide (yellow-fleshed kiwifruit *A. chinensis* var. *chinensis* ‘Hort16A’), Psa tolerance became one of the main screening and selection traits (Beatson et al., 2012; Debenham et al., 2013; Wang et al., 2019). This resulted in the categorization of numerous *Actinidia* species, cultivars and progeny of novel crosses in terms of their susceptibility to Psa (Datson et al., 2015; Hoyte et al., 2015; Nardozza et al., 2015; Wang et al., 2019; Thomidis et al., 2021; Wang et al., 2023a).

As a result of this joint effort, susceptible cultivars are being gradually replaced by more tolerant ones, including var. *chinensis* ‘Gold3’, developed by the breeding program from The New Zealand Institute for Plant & Food Research Limited and the Zespri Group Limited (Ashrafzadeh and Leung, 2019). Nevertheless, there is still an enormous genetic wealth in *Actinidia* spp. germplasm to be explored by, *e.g.*, introgression breeding (Huang and Liu, 2014). Introgressions from the wild *A. eriantha* were firstly aimed at desirable peelability to increase convenience in fruit consumption (Atkinson et al., 2009). Later, *A. eriantha* was used as the male parent in the crossing with *A. chinensis* var. *chinensis* for the commercial release of ‘Jinyan’ (Huang and Liu, 2014), which, apart from other agronomically interesting traits, also showed lower susceptibility to Psa (Lei et al., 2015). Several other species have been pointed as promising breeding material to improve Psa tolerance, including *A. rufa*, *A. kolomycta*, *A. tetramera*, *A. macrosperma*, *A. polygama, A. arguta, A. valvala*, *A. latifolia* and *A. callosa* (Lei et al., 2015; Kisaki et al., 2018; Wang et al., 2019; Wang et al., 2020).

Another aspect to explore is the large variation in ploidy within the genus *Actinidia*, which includes diploids, tetraploids, hexaploids, heptaploids and octaploids species (Huang et al., 2004; Zhang et al., 2017; Asakura and Hoshino, 2018). Higher ploidy has been associated with higher tolerance to Psa (Cotrut et al., 2013; Wang et al., 2020; Wang et al., 2023a). Disease incidence was observed to be higher in diploid and tetraploid *A. chinensis* var. *chinensis* genotypes, whereas in *A. arguta* diploid individuals registered higher susceptibility to Psa and tetraploid individuals showed a lower degree of susceptibility, which was similar to hexaploids of ‘Hayward’ (Cotrut et al., 2013).

Since plant pathogens evolve and their pathogenicity is also influenced by climate change (Anderson et al., 2020), it is important to better explore the plant morpho-physiological, biochemical and molecular mechanisms associated with Psa tolerance. Examples of genotype-dependent traits linked with Psa tolerance include the differential action of enzymes involved in the antioxidant system (*e.g.*, phenylalanine ammonia-lyase, peroxidase and catalase), the sucrose metabolism, functioning of lenticels, density, and size of trichomes, lignification processes, density of stomata and leaf size (Li et al., 2005; Nunes da Silva et al., 2020; Gu et al., 2021; Wang et al., 2023b). All these features should be considered when breeding for resistance to Psa. Furthermore, adequate criteria must be considered in screening for tolerance to Psa, and this aspect is, so far, poorly understood. The extension of lesions in canes, callus formation or assessment of bacterial proliferation within tissues have been considered reliable criteria for assessing susceptibility to Psa (Hoyte et al., 2015; Nardozza et al., 2015; Wang et al., 2019), whereas visual leaf symptoms, *per se*, might be misleading (Wang et al., 2019). Frequently, traditional breeding techniques are expensive, lengthy, and imprecise (Borrelli et al., 2018; Wang et al., 2018c). Techniques of random genetic modification fusing mutagenic agents (*e.g*., ethyl methanesulfonate) are still applied in developing new genotypes, including *Actinidia* cultivars (Sartori et al., 2015). However, the process is very laborious, involving the need to analyze the genetic changes that could be incorporated into elite lines. The more recent possibility of introducing precise modifications into the genome of an organism, through gene-editing, is a major opportunity being used in state-of-the-art plant breeding (Dong and Ronald, 2019), but still greatly unexplored in *Actinidia* spp.

The Clustered Regularly Interspaced Short Palindromic Repeats (CRISPR)/CRISPR-associated protein 9 (Cas9) system is the most recent gene-editing technique revolutionizing biotechnology and has already been applied in numerous crop species to enhance their tolerance against pests and pathogens (Peng et al., 2017; Fister et al., 2018; Ortigosa et al., 2019). Ortigosa et al. (2019) edited the gene in tomato that expresses a co-receptor which is the target of coronatine produced by *Pseudomonas syringae* pv. *tomato*. Since the bacterium uses this phytotoxin to promote leaf colonization through stomata opening (as well as by Psa biovars, but not biovar 3 (Sawada and Fujikawa, 2019)), the gene-editing technique allowed to repress that process, resulting in increased resistance of tomato to the causal agent of the bacterial speck (Ortigosa et al., 2019).

Despite these promising results, the applicability and precision of the CRISPR/Cas9 technology seem to depend on the efficacy of the CRISPR/Cas9 complex (*e.g.*, the combination of the single-guide RNA with the Cas9 protein). Whereas the results have been remarkable for some plant species, it has been less satisfactory for others (Wang et al., 2018c). Nonetheless, three recent studies have shown practical applications of the technology in *Actinidia* (Wang et al., 2018c; Varkonyi-Gasic et al., 2019; Michelotti et al., 2022). Varkonyi-Gasic et al. (2019) edited two *CENTRORADIALIS* (*CEN*)-like genes from *A. chinensis*, namely *AcCEN* and *AcCEN4*, which are thought to be involved in the regulation of plant maturity, architecture and flowering (Voogd et al., 2017). The authors utilized the CRISPR/Cas9 technique with two polycistronic PTG cassettes bound to promoters from *Arabidopsis* sp., to introduce modifications in target sites of those genes, producing smaller and more compact *A. chinensis* plants, with increased precocity (Varkonyi-Gasic et al., 2019). Wang et al. (2018c) were able to optimize gene-edition in *Actinidia* by developing an alternative strategy to CRISPR/Cas9. This strategy consisted in designing a PTG to target the kiwifruit *Phytoene desaturase* (*AcPDS*) gene in different sites and, comparing the PTG/Cas9 gene-editing approach with CRISPR/Cas9, the authors showed a remarkable 10-fold higher target-editing efficiency, thus demonstrating optimization of gene-editing in kiwifruit. Nevertheless, only recently the CRISPR technology was applied aiming at increased kiwifruit tolerance to Psa. In particular, a set of CRISPR vectors were developed for editing a gene belonging to the APETALA2/Ethylene Response Factor (AP2/ERF) transcription factors in a selection of *A. chinensis* var. *chinensis* plants (Michelotti et al., 2022). Given the involvement of AP2/ERF transcription factors in regulating several plant physiological processes, including defense response to both biotic and abiotic stressors (Gu et al., 2017; Bai et al., 2021), the approach might lead to Psa tolerant mutants employing, for example, differential ethylene and ABA regulation (Gunaseelan et al., 2019). However, validation of increased Psa tolerance among the developed *Actinidia* mutants is still lacking (Michelotti et al., 2022).

Nowadays, there are several resources available to assist in the use of gene-editing of plants. They include online tools such as the CRISPRCasFinder and computer programs for Cas designing (*e.g*., Cas-Designer) or off-target identification using machine-learning approaches (*e.g*., VARSCOT) (Park et al., 2015; Couvin et al., 2018; Wilson et al., 2019). Moreover, in the specific case of kiwifruit, databases with genomic information on *Actinidia* spp. have been made available (Wang and Lin-Wang, 2007; Yue et al., 2020). However, several operational constraints related to the commercial release of genetically modified organisms still occur, limiting the use of gene-editing applications, particularly in regions with stricter regulations as *e.g*., Europe (Callaway, 2018).

The knowledge of the genes to be targeted in plant breeding programs is the fundamental basis for the development of Psa-tolerant kiwifruit genotypes. In this respect, much remains to be unraveled about the genetic bases underlying tolerance traits, but progress has been made with kiwifruit genome sequencing (Huang et al., 2013; Tang et al., 2019) and greater knowledge on functional genomics of kiwifruit plants infected by Psa (Wang et al., 2018b; Nunes da Silva et al., 2019; Tahir et al., 2019; Nunes da Silva et al., 2020; Nunes da Silva et al., 2022a).

### 2.3 Plant elicitors

One alternative strategy to using conventional pesticides relies on inducing plant resistance with elicitors (also known as defense inducers, plant activators or priming agents) (Ramírez-Carrasco et al., 2017; Oliveira et al., 2019). Until now, numerous chemical compounds have been reported as plant elicitors, including phytohormones and their analogues, carbohydrate polymers, lipids, glycoproteins, vitamins and polysaccharides (Bektas and Eulgem, 2015; Mauch-Mani et al., 2017; Tripathi et al., 2019) as well as beneficial elements such as silicon (Bokor et al., 2021). For instance, salicylic acid (SA) and its functional analogues benzothiadiazole (BTH) and b-aminobutyric acid (BABA) are among the most studied for their action as broad-spectrum defense inducers through systemic acquired resistance (SAR) (Tripathi et al., 2019). The most used BTH in kiwifruit protection is acibenzolar-*S*-methyl (ASM), commercialized as Bion^®^ or Actigard^®^. Several works have shown the effectiveness of ASM in controlling Psa in *Actinidia* spp., despite the effect being genotype dependent. For example, Cellini et al. (2014) showed that *A. chinensis* var. *deliciosa* plants with lower Psa incidence were more prone to form necrotic spots, deposit callose and have superoxide bursts after ASM application, while the same was not observed in *A. chinensis* var. *chinensis*. Nevertheless, the protective effect of ASM was maintained for several weeks in both species (Cellini et al., 2014). Additionally, Wurms et al. (2017) observed that ASM significantly reduced the lesion length of Psa infection by 50%, indicating that primed plants could respond more strongly to the infection. Interestingly, elicitation of the ethylene (ET) and jasmonic acid (JA) pathways were associated with the worsening of disease incidence, most likely owed to SA pathway suppression and promotion of stomatal opening, which facilitates Psa invasion into the host tissues (Bektas and Eulgem, 2015; Michelotti et al., 2018; Brunetti et al., 2020; Nunes da Silva et al., 2021a).

The application of ASM has also shown a synergistic protective effect with MBCAs (de Jong et al., 2019) and in providing vine protection during the post-harvest stage (Reglinski et al., 2023). Enhancement of plant defense capacity during this stage is particularly important due to the occurrence of plant wounding during fruit harvest. Notwithstanding, co-application of ASM and copper demonstrated capacity to upregulate defense genes (using copper treatment as control) such as *Benzyl alcohol dehydrogenase* (*BAD*), *Downy mildew resistance 6* (*DMR6*), *NIM-interacting protein 2* (*NIMIN2*) and *WRKY70i*, associated to the SA pathway and to the encoding of pathogenesis-related (PR) proteins (Reglinski et al., 2023). The resulting state of “alertness” of the kiwifruit vines provides enhanced defense responses, when plants are challenged with microbial phytopathogens such as Psa (de Jong et al., 2019; Reglinski et al., 2023).

BABA is a plant metabolite that tends to accumulate in sites of bacterial or fungal infection, with potential for controlling pathogens and pests in several plant species (Baccelli and Mauch-Mani, 2016; Li et al., 2019). In kiwifruit, Brunetti et al. (2020) presented promising results in a leaf disk assay, with BABA showing Psa symptom reduction, which might have been due to a direct bactericidal effect.

Apart from SA-analogues, chitosan is one of the most studied elicitors and has already been included in commercial products for field applications in kiwifruit orchards (Collina et al., 2016; Brunetti et al., 2020). This compound is believed to act as bacteriostatic, affecting the biochemistry of the bacterial surface (Shanmugam et al., 2016), and may also hamper the pathogen’s access to nutrients and minerals (Beatrice et al., 2017). Chitosan-elicited kiwifruit plants have shown enhanced expression of genes encoding PR proteins after Psa inoculation (Beatrice et al., 2017). Field efficacy to control Psa was demonstrated in a three-year trial with *A. chinensis* var. *deliciosa* ‘Hayward’, where leaf spots, twig wilting, and the number of exudates were significantly reduced due to chitosan application (Scortichini, 2014). Numerous positive attributes have been pointed to chitosan, including its broad activity spectrum, biodegradability and biocompatibility (Scortichini, 2014; Beatrice et al., 2017; Yang and Zhong, 2017); however, an important drawback for field application is its insolubility in water. To overcome this, several water-soluble chitosan derivatives have been obtained. In addition, several chitosan derivatives have been reported as having higher antibacterial activity than chitosan itself, especially against Gram-negative bacteria, possibly due to differences in the bacteriostatic effect and interference with the pathogen homeostasis (Yang and Zhong, 2017).

Plant elicitors are a viable alternative to conventional pesticides, and we believe that there will be an increment in their use in the coming years. Notwithstanding, further studies are required for optimizing elicitors´ use regarding application timings, methods and doses.

### 2.4 Emerging molecules and compounds

#### 2.4.1 Essential oils and other bioactive compounds

Plants produce a wide diversity of substances used for signaling processes and defense against invaders, which include numerous bioactive molecules/compounds present in plant extracts (*lato sensu*) and, more specifically, in plant EOs (Song et al., 2016; Mori et al., 2019; Sciubba et al., 2019). The antibacterial activity of plant extracts has been attributed *e.g.* to the alterations in the permeability of pathogen cell surface, ultimately leading to the loss of intracellular contents and the death of the microorganism, and affecting its quorum sensing mechanisms (Chouhan et al., 2017; Pucci et al., 2018).

Activity against Psa and other pseudomonads were found in extracts from plants from the Liliales and Urticales orders, which inhibited bacterial multiplication for, at least, two weeks after leaf spraying (Balestra, 2007). Antimicrobial activity against Psa was also demonstrated for extracts and EOs from plants belonging to the families Myrtaceae (*Pimenta* spp., *Melaleuca* spp. and *Syzygium aromaticum*), Lauraceae (*Cinnamomum* spp. and *Laurus* spp.), Lamiaceae (*Mentha suaveolens*, *Rosmarinus* spp., *Salvia* spp., *Monarda* spp., *Thymus vulgaris* and *Origanum vulgare*), Poaceae (*Phyllostachys heterocycla f. pubescens*), Euphorbiaceae (*Sapium baccatum*), Amaryllidaceae (*Allium sativum*), Theaceae, Hypericaceae (*Hypericum perforatum*), Polygonaceae (*Polygonum cuspidatum*) and Vitaceae (*Vitis vinifera*) (Song et al., 2016; Vavala et al., 2016; Mattarelli et al., 2017; Vu et al., 2017; Pucci et al., 2018; Lovato et al., 2019; Mori et al., 2019; Simonetti et al., 2020). Until now, among the most effective EOs against Psa, as indicated by laboratory trials, are those rich in phenolic compounds, stilbenes, xanthones, olefinic compounds, cinnamaldehyde and tannins (Song et al., 2016; Vu et al., 2017; Mori et al., 2019; Simonetti et al., 2020). Song et al. (2016) showed the inhibitory effect against Psa of the constituents eugenol, methyl eugenol and estragole, from *Pimenta* spp. EO, and cinnamaldehyde, from *Cinnamomum cassia* EO. Activity against Psa and other bacteria was also demonstrated *in vitro* for several constituents of EOs isolated from the medicinal plant *Sapium baccatum*, including, methyl gallate, corilagin, tercatain, chebulagic acid, chebulinic acid, quercetin 3-O-α-L-arabinopyranoside and gallic acid (Vu et al., 2017).

Lovato et al. (2019) demonstrated the suppression of Psa virulence traits after exposure to green tea extracts (*Camellia sinensis*) by reducing pathogen motility and biofilm production. Despite the promising ability of EOs to inhibit Psa, their composition is strongly influenced by the plant growth conditions, chemotype, plant age and organ used (Pucci et al., 2018). Moreover, the extraction method is decisive relatively to what can be maintained or lost in EOs extract preparation (Mori et al., 2019). For example, Mori et al. (2019) showed that the antimicrobial activity of EOs from *P. h.* f. *pubescens* against Psa and other plant pathogens was considerably higher for EOs extracted by super-heated steam, as compared with EOs extracted with ethanol or hot water. In addition, Mattarelli et al. (2017) observed marked differences in the composition of EOs extracted from the closely related species *Monarda didyma* and *M. fistulosa*, not only in terms of constituent detected but also in their relative abundance. Despite this, their activity against Psa was similar, presumably because of higher content of carvacrol, observed in the EO with less thymol. Later, it was demonstrated, *in vitro*, that the antimicrobial capacity of carvacrol against Psa (including inhibition of Psa growth and biofilm formation) could be enhanced when combined with phages (Ni et al., 2020). Additionally, EOs can act synergistically, which might result in some cases in much lower doses needed for their efficacy against pathogens (Vavala et al., 2016), although little is known about the interactions leading to synergism or antagonism between EOs or their constituents (Chouhan et al., 2017).

Despite the clear advantages of using EOs in plant protection, phytotoxicity, difficulties in the uniformization and perishability of the products, low solubility and chemical instability, limit their inclusion in commercial formulations (Rossetti et al., 2017; Pucci et al., 2018). Nanotechnology can address some of those limitations. This was demonstrated by microencapsulating gallic acid and ellagic acid to control bacterial pathogens in kiwifruit plants (Rossetti et al., 2017). Nevertheless, the antimicrobial activity of integral EOs and their constituents, combined or applied alone, should be subject to further investigation and tested in the field. As these limitations are overcome and further research is developed, more EO-based products are very likely to emerge in the market soon.

Propolis is rich in EOs and in several bioactive compounds, including phenols, aromatic acids, diterpenic acids, aldehydes, monoterpene, amino acids, steroids, and inorganic compounds (Bulman et al., 2011; Gavanji et al., 2012; Pobiega et al., 2019). Despite major differences observed in the composition of different types of propolis, they are consistently referred as having a broad range of biological activities against bacteria (Ordóñez et al., 2011; Jaski et al., 2019), but studies on their use against Psa are still lacking.

Several bioactive compounds originated from plants have been investigated individually. One example includes forsythoside A, a phenylethanoid glycoside with antimicrobial properties obtained from the medicinal plant *Forsythia suspensa* (Oleaceae family) (Yuan et al., 2022). This compound has shown lytic capacity against Psa, as well as effectiveness in preventing biofilm formation, reducing swimming motility and affecting Psa biochemical processes (*e.g*., reduction of Psa IAA synthesis and interfering with the expression of virulence genes) (Pei et al., 2022). *In planta*, this resulted in decreased KBC symptoms in *A. chinensis* var. *deliciosa* ‘Hongyong’ potted plants (Pei et al., 2022).

Plant elicitation may also trigger the synthesis of potent bioactive compounds in plants. For example, root extracts from the xanthone-rich plant *Hypericum perforatum* elicited with chitosan oligosaccharides showed higher activity against Psa, when compared with extract from roots of non-elicited plants (Simonetti et al., 2020).

#### 2.4.2 Antimicrobial peptides

Antimicrobial Peptides (AMPs) are short-chain molecules constituted of sequences of amino acids (typically between two and fifty amino acids) (Zhang et al., 2023b). Natural AMPs are universally distributed in living organisms, as part of the defense system of bacteria, protozoans, fungi, plants and animals (Srivastava et al., 2021). These biomolecules present microbiocidal and microbiostatic activity, also being able to modulate immune responses exclusively against prokaryotes (Kumar et al., 2018; Chen et al., 2021). For plant protection, there is a number of AMP-based products currently under development, and a few AMP-based commercial products have already been released to the market (Zhang et al., 2023b). However, to the best of our knowledge, there are no commercially available AMP-based products to be used against Psa.

The bactericidal capacity of AMPs is based on two main modes of action, namely including cell lysis through disruption of bacterial membranes and entrance inside the bacteria for interacting with intracellular components (Benfield and Henriques, 2020). Despite much remains to be unraveled about their functioning, it is known that the antimicrobial capacity of AMPs is related to the cationic activity of amino acids such as lysine and arginine, as well as to the hydrophobicity of amino acids like leucine, isoleucine, valine, phenylalanine and tryptophan targeting the cell membrane of the target microbe, and to the ratio between hydrophilic and hydrophobic residues or domains present in AMPs, a feature known as amphipathicity (Hale and Hancock, 2007; Kumar et al., 2018; Srivastava et al., 2021). Moreover, it has been pointed out the importance of the spatial arrangement of AMPs, establishing a relationship between the localization of hydrophobic, cationic or polar residues within AMPs and their antimicrobial capacity (Nguyen et al., 2011; Chen et al., 2021).

The AMPs have been included in several databases, such as the Antimicrobial Peptide Database (APD; https://aps.unmc.edu/) among others listed elsewhere, for disseminating their applicability, predicting their functioning and supporting the design of novel AMPs (Wang, 2015).

The use of AMPs in the control of pathogens shows several advantages such as their abundance of sources, the possibility of designing AMPs, rapid antimicrobial action, the capacity of degrading biofilms, low likelihood of promoting microbial resistance and efficacy in controlling metabolically inactive microbes (Han et al., 2021). The main disadvantages include their *in vivo* activity being diminished by ionic interactions, degradation by proteases and peptidases, the possibility of causing cytotoxicity, low-specificity, which may cause biological imbalances, and high cost in their production mainly due to the extraction and purification processes (Jung and Kang, 2014; Holaskova et al., 2015; Han et al., 2021). Nevertheless, these drawbacks have been gradually overcome, with existing AMPs serving as templates for designing novel molecules of enhanced antimicrobial capacity, with better stability, increased specificity, and lower cytotoxicity (Eckert et al., 2006; Jang et al., 2012). Candidate AMPs are screened through a trial-and-error approach, and synthetic AMPs with improved characteristics are constructed with modifications, multimerizations and inclusions of artificial amino acids and peptides (Zeitler et al., 2013; Kai et al., 2018; Gong et al., 2019; Chen et al., 2021). Regarding Psa, the first steps in the use of AMPs against this bacterium were made almost one decade ago. Cameron et al. (2014) reported short cationic AMPs with activity against Psa *in vitro* (the dodecapeptide amide, KYKLFKKILKFL-NH_2_, and the hexapeptide amide, WRWYCR-NH_2_), which showed effectiveness in controlling Psa comparable to that of streptomycin. The authors also showed that conjugation with the toxic moiety 5-nitro-2-furaldehyde (NFA) may be used for designing novel AMPs against Psa, whereas, in this case, acylation diminished the antimicrobial activity of the tested AMPs. Later, analogues of the cyclic lipopeptide battacin, produced in nature by the soil-borne bacterium *Paneibacillus tianmunesis*, have demonstrated enhanced antimicrobial activity if their synthesis includes linearization of the original structure. The most active was further tested *in vitro* for its ability to act against Psa, proving to be highly effective either in preventing biofilm formation or eradicating preformed biofilms (Zoysa et al., 2015). Activity against Psa and other phytopathogens was also demonstrated for a collection of 36 linear lipopeptides from the cecropin A-melittin hybrid undecapeptide (BP100). In general, it was observed that lipopeptides bearing a hexanoyl chain were more effective against both Psa and *P. syringae* pv. *syringae* (Oliveras et al., 2018). Mariz-Ponte et al. (2021) performed antibiograms for selecting candidate AMPs for further analysis against Psa. This resulted in the selection of three AMPs for conducting an *in vitro* assessment of their activity against eight Psa strains, utilizing the well-studied BP100 as a reference, and a flow cytometry technique to inspect bacterial membrane integrity, during the exposure to the tested AMPs. The authors suggest CA-M and 3.1 peptides as promising molecules for mixing with BP100 due to their higher bacterial permeabilization capacity when compared with BP100 (Mariz-Ponte et al., 2021). Hence, this constitutes an example of the potential of combining AMPs with different modes of action, aiming at a synergistic effect of these biomolecules against plant pathogens. More recently, the AMP Jelleine-Ic has shown effective control of Psa both *in vitro* and *in vivo*. This AMP showed ability to alter the bacterial membrane integrity, with strong consequences at the intracellular level in Psa. Moreover, Jelleine-Ic downregulated bacterial genes related to replication and damage recovery, which ultimately resulted in alleviating KBC symptoms in kiwifruit leaves (Zhang et al., 2023a).

Considering the natural sources of AMPs and the structural and biochemical modifications that can be performed in the synthesis of novel peptides, the possibilities are enormous. Moreover, despite many research studies on the activity of AMPs, *in planta* trials are still lacking. Limitations such as cytotoxicity, poor *in vivo* performance, instability of the substances and production costs are still major obstacles, concerning the application of AMPs against plant pathogens (Tang et al., 2018). These aspects have been tackled through peptide engineering, with production costs being gradually reduced because of advances in their synthesis, including the application of recombinant techniques using *E. coli* and yeast (Benfield and Henriques, 2020). The growing list of candidate AMPs to be used in the control of Psa points out to emergence of AMP-based commercial products to be used in KBC mitigation in the forthcoming years.

### 2.5 Microbial biological control agents

In recent years, the use of MBCAs for suppressing phytopathogens has become one of the dominating topics in plant disease management, with strong research and development of new Plant Protection Products (PPP) and many commercial products already in the market containing (beneficial) fungi, bacteria, and phages, as well as several bioactive compounds obtained from them (Fira et al., 2018; Pereira et al., 2021; Córdova et al., 2023). The great potential of MBCAs for controlling plant pathogens is based on several ecological characteristics, which confer a variety of action modes against their target, as recently reviewed by other authors (Köhl et al., 2019; Córdova et al., 2023).

#### 2.5.1 Phages

Phages (or bacteriophages) are viruses that exclusively infect bacteria and whose genome consists of single-stranded or double-stranded DNA or RNA (Liu et al., 2022). They are the most abundant type of biological entity known to date, being virtually found in all environments (Di Lallo et al., 2014; Frampton et al., 2014). Despite being seen as promising agents for controlling phytopathogens since 1926, their application in agricultural settings remained minor for several decades, with phages mostly used for typing plant-infecting bacteria (Sulakvelidze and Barrow, 2005). However, this scenario has been changing in recent years, because of the need for providing suitable alternatives to antibiotics and copper-based compounds in plant disease management.

Phage taxonomy traditionally relied on morphological traits, but modern genomic tools have shifted the classification to a genome-driven approach (Chibani et al., 2019). This method enhances phylogenetic accuracy and aligns with phages’ mode of action, categorized into lysogenic and lytic cycles (Kering et al., 2019). Lysogenic phages integrate into bacterial genomes, delaying host disruption until triggered by environmental cues, while lytic phages swiftly destroy hosts upon injection (Vu and Oh, 2020; Liu et al., 2022). Lytic phages, preferred for plant protection due to higher efficacy and biological safety, are crucial in selecting phages for plant disease management. Key considerations include avoiding genetic transfer to hosts, efficient pathogen control (*e.g*., time of death), and specificity (Di Lallo et al., 2014; Frampton et al., 2014; Flores et al., 2020).

The success of phages in controlling several phytopathogens had led to the emergence of several phage-based commercial products, but none of them has been specifically developed to control Psa (Pereira et al., 2021; Wagemans et al., 2022). Nevertheless, an extensive list of candidate phages has been made available following several trials where their efficacy against Psa has been demonstrated both *in vitro* (Pinheiro et al., 2019) and *in vivo* (Flores et al., 2020). Active phages against Psa have been isolated either from healthy or Psa-infected *Actinidia* spp., as also from other sources such as the orchard environment, water bodies, or even from public sewage (Di Lallo et al., 2014; Yu et al., 2016; Park et al., 2018; Yin et al., 2019; Flores et al., 2020; Liu et al., 2021; Ni et al., 2021; Bai et al., 2022; Zhang et al., 2022). After an exhaustive screening of phage-host combinations, Frampton et al. (2014) selected 24 from more than 250 phages with activity against Psa, including phages with both narrow and broad host ranges. From this assessment using Psa strains and other pseudomonads (collected in kiwifruit orchards from several countries), the authors concluded that most of the phages against Psa belong to the Caudovirales order, have narrow interspecific host range and, presumably, use several strategies in the infection process. Nevertheless, this gives a gigantic basis of work to plant researchers, as it has been estimated that about 96% of phages fall into the Claudovirales order, which is further categorized into three families, depending on their tail structure: Myoviridae (contractile tail), Siphoviridae (non-retractable long tail) and Podoviridae (short tail) (Liu et al., 2022).

The commercially available RNA phage ϕ6 (from DSMZ Braunschweig, Germany) from the Cystoviridae family (Mindivirales order), originally applied in other pathogenic pseudomonads, was recently tested against Psa (Pinheiro et al., 2020). This phage showed efficacy in killing the bacteria both *in vitro* and *ex vivo*, using artificially inoculated leaves from *Actinidia* sp. (variety not disclosed) and two Psa strains belonging to biovar 3 (Pinheiro et al., 2020). Another study demonstrated enhanced capacity of a combination of two RNA phages from the Siphoviridae family with two DNA phages from the Podoviridae family in controlling Psa *in planta*, utilizing artificially inoculated *A. chinensis* var. *rufopulpa* seedlings (Bai et al., 2022). In particular, the authors observed stronger lytic capacity and prolonged growth of the phages within the mixture, when compared to their action alone (Bai et al., 2022).

Ni et al. (2021) assessed the lytic spectrum of the phage PN09, from the Myoviridae family, using 29 Psa strains and other five bacterial species (including *P. aeruginosa*) *in vitro*. This phage showed high specificity, causing lysis in all tested Psa strains while being innocuous to the other tested species. Furthermore, the endolysins produced by PN09 (LysPN09) showed lytic activity of about 65% (assessed through turbidimetry) when combined with ethylenediaminetetraacetic acid (EDTA) (Ni et al., 2021). Phage endolysins are crucial in bacterial virulence, as they degrade the peptidoglycan layer. In Gram-negative bacteria like Psa, effective disruption of the outer membrane and peptidoglycan layer during phage infection requires collaboration with adjuvant proteins, including holins, pinholins, and spanins (Gontijo et al., 2021). In fact, the lack of bacterial resistance against phages is largely due to the lytic action of endolysins targeting a strongly conserved structure such as the peptidoglycan layer among bacteria, making it hard for these microorganisms to evolve alternative mechanisms to defend themselves from phages (Gontijo et al., 2021). However, the absence of the adjuvant proteins in *ex vivo* applications of endolysins makes it necessary the use of membrane permeabilizers such as EDTA, citric acid, malic acid, lactic acid, benzoic acid or acetic acid, so endolysins can effectively reach the peptidoglycan layer of their hosts (Oliveira et al., 2016).

Recently, the efficacy of phage therapy in mitigating KBC was validated *in planta*, using several phages from the Podoviridae family (Flores et al., 2020; Song et al., 2021; Fiorillo et al., 2023). Flores et al. (2020) showed Psa load reduction and fewer KBC symptoms after the application of a phage cocktail applied to two-year-old ‘Hayward’ plants artificially inoculated with Psa. This cocktail contained four phages, which were selected for their lytic activity against Psa and tolerance to a wide range of temperature (4-37 °C), pH (4-7) and exposure to solar radiation (up to 60 min). Nevertheless, the putative synergism among different phages must be assessed on a case-by-case basis. For example, Song et al. (2021) observed that the phage PPPL-1 significantly reduced the KBC symptoms in six-week-old grafted plants grown in pots (with ‘Hayward’ and ‘Haehyang’ as rootstock and scion, respectively), while not confirming enhanced antibacterial capacity of a cocktail combining this phage with two other with known lytic activity against Psa. More recently, the phage φPSA2 (characterized in Di Lallo et al. (2014)), also from the Podoviridae family, demonstrated capacity to counteract Psa colonization capacity inside plant tissues and to reduce KBC symptoms in kiwifruit plants (Fiorillo et al., 2023). Hence, combinations of phages must be tested for their suitability to incorporate cocktails with increased antibacterial action. This is because their high specificity often restricts their action at the bacterial strain level (Hyman and Abedon, 2010). In fact, this aspect may represent either an advantage or an inconvenience. On the one hand, their high specificity confers biological safety but, on the other hand, this feature limits their antimicrobial action and may constitute a strong limitation to the market value of phage-based products, hampering their upscaling under multiple-target scenarios. For these reasons, to date, no commercial phage products have been approved to be used against Psa.

#### 2.5.2 Fungi and bacteria

The modes of action of MBCAs are related to three categories of antagonistic interactions between MBCAs and plant pathogens: direct antagonism, mixed-path antagonism and indirect antagonism (Wachowska et al., 2017). Direct antagonism involves killing the pathogenic target through hyperparasitism/predation, whereas mixed-path antagonism involves suppression mechanisms through the production of antibiotics, lytic enzymes, bacteriocins, pathogen-inhibiting volatile compounds and siderophores. In turn, indirect antagonism is related to the induction of host resistance and/or to competition for ecological niches against pathogens (Tontou et al., 2016a; Tontou et al., 2016b; Wachowska et al., 2017; Compant et al., 2019; Gimranov et al., 2022).

An increasing list of beneficial fungi and bacteria applied to control Psa in kiwifruit orchards has been made available in recent years, including the fungi *Aureobasidium pullulans*, *Trichoderma* spp. and *Ulocladium oudemansii* and the bacteria *Bacillus subtilis*, *B. amyloliquefaciens* subsp. *plantarum*, *P. fluorescens* and *Pantoea agglomerans* (Stewart et al., 2011; Hill et al., 2015; Woodcock, 2016; Hoyte et al., 2018; de Jong et al., 2019; Rheinländer et al., 2021; Biondi et al., 2022). The kiwifruit ecosphere has been utilized as a source of microorganisms with antagonistic action against Psa. For example, Kim et al. (2019) performed a stepwise screening of bacteria for their activity against Psa, beginning with about three thousand isolates collected from rhizosphere soil, branch sap and pollen from *A. chinensis* var. *deliciosa* ‘Deliwoong’. The authors selected the bacteria *Streptomyces* sp. (undisclosed species), *Streptomyces racemochromogenes* (both collected from rhizosphere) and *S. parvulus* (collected from pollen) for their strong *in vitro* activity against Psa, and subsequent confirmation *in planta*, where these species demonstrated to reduce KBC symptoms in 12-week-old ‘Deliwoong’ plants. Also noteworthy was their capacity of colonizing both roots and leaves, opening good perspectives for their application as PPP (Kim et al., 2019). Several pseudomonads extracted from kiwifruit plants have also been tested as MBCAs against Psa. For example, *P. putida* and *P. poae* extracted as epiphytes from ‘Hayward’ leaves collected from kiwifruit orchards, have demonstrated their capacity to antagonize Psa on flower surfaces due to a fast epiphytic colonization. The authors also showed that the application of these MBCAs could provide epiphytic protection when applied under mild temperatures (10 °C), as this was tested in the experiment to mimic those which represent a higher risk of Psa infection in the field (Correia et al., 2022). In another study, the endophyte *P. synxantha*, isolated from *A. chinensis* var. *chinensis*, was proposed for further investigation due to its antagonistic activity against Psa, possibly resulting from the presence of genes involved in the synthesis of nonribosomal peptide synthetases, which may antagonize this phytopathogen (Tontou et al., 2016a; Tontou et al., 2016b). It was demonstrated that the endophytic bacterium *P. bijieensis* XL17 (*P. corrugata* subgroup), isolated from a crown gall of a rape plant, and its cell-free culture filtrate could antagonize Psa in *in vivo* trials (detached leaves) (Ali et al., 2022). Scanning Electron Microscopy (SEM) and Transmission Electron Microscopy (TEM) techniques were applied to show that *P. bijieensis* and its filtrate were able to destroy cells from this pathogen in an antagonistic action in which the phenolic polyketide 2,4-diacetylphloroglucinol (DAPG) and lipopeptides are likely involved (Ali et al., 2022). Therefore, the potential of using MBCAs goes beyond their direct action against the target phytopathogen, as several bioactive compounds can be obtained from them. For example, isobenzofuranones and isocoumarins obtained from the fungus *Paraphaeosphaeria sporulosa* and imidazole alkaloids obtained from *Fusarium tricinctum*, both fungal species extracted epiphytically from healthy kiwifruit plants, have demonstrated capacity to inhibit the growth of Psa in plate assays (Chen et al., 2022; Ma et al., 2022; Ma et al., 2023). Other bioactive compounds extracted from the fungus *Zopfiella* sp. obtained from kiwifruit plants include 3-decalinoyltetramic acid derivatives (Yi et al., 2021). In addition, the kiwifruit endophytic fungus *Bipolaris* sp. was found to have terpenoids (including sesquiterpenoids, sesterterpenoids and analogues) and xanthones with antimicrobial activity against Psa (and other phytopathogens), notwithstanding being less potent in inhibiting Psa growth than streptomycin (used as control, in plate assays) (Yu et al., 2022a; Yu et al., 2022b).

Extracts from four marine bacteria belonging to the phylum Planctomycetes, namely *Alienimonas chondri*, *Rhodopirellula rubra*, *Rubinisphaera brasiliensis* and *Novipirellula caenicola* obtained from the macroalgal biofilm of *Gracilaria bursa-pastoris* showed bacteriostatic effect against Psa and other phytopathogenic pseudomonads in a plate assay, whereas extracts from three bacterial species from the phylum Actinobacteria did not show antagonism against the tested targets (Gimranov et al., 2022). Furthermore, lactic acid bacteria (*e.g*., *Lactobacillus plantarum*) have also been considered a promising group because of their production of acidifying metabolites (organic acids), antimicrobial peptides (*e.g*., bacteriocins) and other bioactive compounds (Daranas et al., 2018; Purahong et al., 2018). Endophytic bacteria from the New Zealand native *Leptospermum scoparium* also showed activity against Psa both *in vitro* and in *A. chinensis* var. *deliciosa* plants, in which endophytes were inoculated through stem wounding and foliar spraying (Wicaksono et al., 2018). A few commercial MBCAs-based PPPs originally conceived for targeting other phytopathogens have been approved for KBC management. This was the case of Amylo-X^®^ (based on *B. amyloliquefaciens* subsp. *plantarum*) and BOTRY-ZenR (based on *U. oudemansii*), after demonstrating the capacity to control Psa in the field, whereas AUREO^®^ Gold (based on *A. pullulans*) and Kiwivax^®^ (Based on *T. atroviride* and *T. virens*) were developed having Psa as main target (Hoyte et al., 2018; Rheinländer et al., 2021; Biondi et al., 2022). However, a full satisfactory level of preventive and curative capacity has not yet been achieved. Low mobility within the plant and the lack of knowledge on the persistence of the inoculated organisms are among the reasons why the long-term efficacy of using MBCAs is still seen with some reservations when considering field applications (Wicaksono et al., 2018). The development of MBCA-based products must go through the laborious path of a thorough comprehension of their mode of action to assure the lack of environmental disturbances related to their use (*e.g*., impact on pollinators). Furthermore, the approval of this type of PPP implicates strict and costly legal processes, which constitute important constraints for the release of new commercial products.

## 3 Overview on the scientific and technological progress of available tools for sustainable KBC management

**Figure 1:**
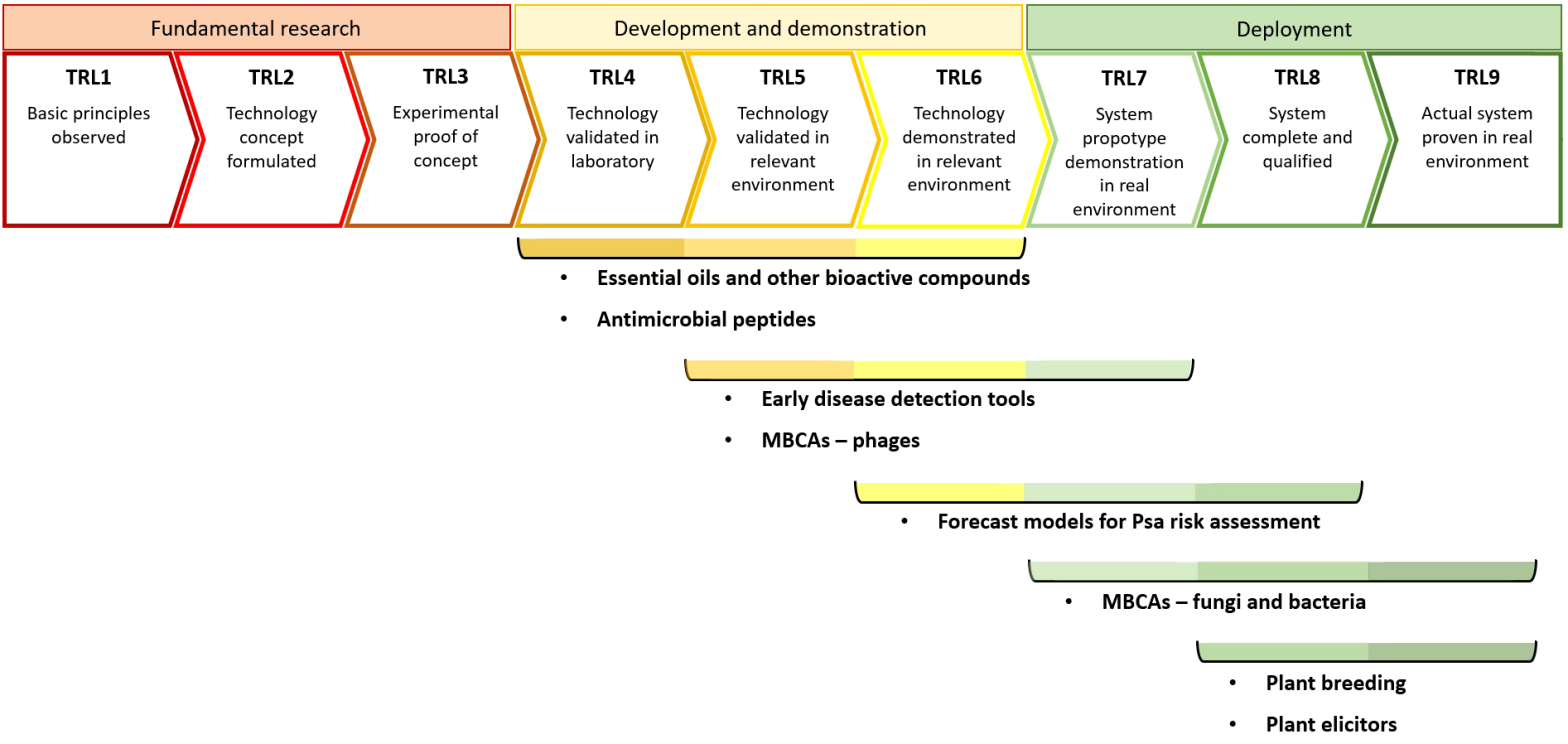
Estimate of the current Technology Readiness Levels (TRLs) of different disease management tools for the mitigation of the kiwifruit bacterial canker. MBCAs, Microbial Biological Control Agents; Psa, *Pseudomonas syringae* pv. *actinidiae*.

Nearly fifteen years after the 2008 Psa outbreak, some of the disease management tools have evolved to the point of their commercial application, whereas other are still at more precocious stages of technological maturity. One of the most commonly used methods for assessing the level of maturity, between technologies that have little in common, consists in the Technology Readiness Level (TRL) model, which is presented in a nine-point system encompassing fundamental research (TRL1-TRL3), development and demonstration (TRL4-TRL6) and deployment of a given technology to a real environment (TRL7-TRL9) (White et al., 2022). **Figure 1** depicts our view on the current technological maturity of each tool discussed above. The diagram was constructed considering three premises: (i) the technological maturity of each tool covered more than one TRL for better reflecting the diversity of developmental status of different molecules or techniques, within each disease management tool applied to KBC; (ii) achieving the validation and demonstration in relevant environment (TRL5 and TRL6, respectively) requires *in vivo* experiments (*i.e*., involving plant material) with a consistent degree of positive results; (iii) the evolution of the tools to the deployment phase (≥ TRL7) requires positive results in managing KBC in real environment (*i.e*., under field conditions), further distinguishing the consistency of results to a qualified level (TRL8) and evolution to the point of deployment of commercial solutions for KBC management (TRL9). Thus, the TRL analysis further evidences the role of plant breeding in a significant, though gradual, mitigation of KBC following the devastating impacts caused by biovar 3 of Psa. Indeed, at the moment of the 2008 outbreak, adequate tools to deal with this highly virulent phytopathogen were not available for the kiwifruit industry to effectively and timely address the KBC problem (Vanneste et al., 2011). Thereafter, the gradual substitution of Psa-sensitive cultivars with more tolerant ones has been consistently contributing to a recovery of the kiwifruit industry. In turn, the precision agriculture tools addressed in the current review (*i.e*., early disease detection tools; forecast models for Psa risk assessment) are still ongoing more precocious stages in the path to deploying effective and consistent solutions for their commercial application for KBC management, accessible to kiwifruit growers (**Figure 1**).

In what regards the other disease management tools, it is clear that the molecules and MBCAs referred in the present work are currently at different stages in the development of novel PPP to counteract KBC. For instance, plant elicitors and fungal and bacterial MBCAs against Psa have already reached TRL9 (commercial application), whereas EOs and AMPs are found at lower TRLs (**Figure 1**). Indeed, a considerable part of these tools are presently ongoing laboratorial screenings, whereas other have been tested *in planta*, inclusively in greenhouse and field trials. **Supplementary Table S1** (**Supplementary Material**) illustrates the diversity of molecules and MBCAs that have been tested for KBC mitigation, with a large part of the studies focusing on plant elicitors, EOs and other bioactive compounds, whereas the utilization of AMPs and phages remain poorly explored in *Actinidia*-Psa. In addition, the screening of novel molecules or MBCAs to be used against Psa is commonly made recurring to plate assays, and *in planta* trials most often utilize foliar spraying as application method in climate chamber, greenhouse or open-field conditions. Regarding the mode of action, there is a preponderance of plant elicitation of the SA-pathway, competition against Psa (in the case of MBCAs), bacteriophagy and antibacterial activity, both related to the bacteriostatic and bactericidal capacity of a number of molecules, mainly from EOs. Several molecules, especially plant elicitors, and MBCAs have already been tested under greenhouse and field conditions. Surprisingly, despite the promising results in relation to the application of EOs or their constituents in laboratory trials, there is a clear lack of studies addressing their testing in greenhouse or field conditions.

In respect to the assessment of the treatments’ efficacy, numerous molecules have shown ability to reduce visual symptoms in plant tissues, mainly in leaves, often subsequently to enhanced plant immune responses, with up-regulation and overexpression of defense-related genes in Psa-infected kiwifruit plants. Some of those studies point-out those responses as being genotype-dependent, further evidencing the necessity to increase the knowledge about susceptibility, tolerance and resistance to Psa of *Actinidia* genotypes. Furthermore, synergistic effects between molecules have been reported, inclusively when combining chemical elicitors (*e.g*., ASM) with fungal MBCAs such as *A. Pullulans* and *Thrichoderma* spp. (Hill et al., 2015; de Jong et al., 2019).

Most often, the *in vivo* antibacterial activity of the molecules has been referred in terms of Psa growth control, not allowing a fully understanding whether the results reflect a bacteriostatic or bactericidal effect against Psa. Nevertheless, studies including the testing of phages refer to Psa load reduction, pointing towards effective bacteriophagy of these microorganisms, belonging mainly to the families Cystoviridae, Podoviridae, Siphoviridae and Myoviridae, which have shown promising groups to explore in screening phages against Psa (Pinheiro et al., 2020; Bai et al., 2022; Liu et al., 2022).

Phytotoxicity and stability of the substances relatively to environmental conditions (*e.g*., temperature, UV radiation, pH) were utilized as criteria for assessing the suitability of several molecules for *in planta* application. Furthermore, the potential threat of pathogens to evolve, becoming more virulent or resistant to existing control methods, further increases the unpredictability in the development and deployment of plant disease management strategies (Colombi et al., 2017; Hemara et al., 2022). Until now, this has been addressed by targeting highly conserved structures of the pathogen. Apart from the need for guaranteeing low probability of bacterial resistance acquisition by Psa, it is also necessary to assure low environmental persistence of the new generation PPP to be used in KBC management. In fact, these two criteria have been the main motivations behind the recommendation of substitution of copper-based compounds in PPPs in the European Union (European Commission, 2018) and, in this sense, the plant elicitors, emerging molecules and MBCAs may render suitable alternatives.

In addition, innovative disease management tools, other than those presented in this literature review, may emerge possibly leveraged by the increasing knowledge about the Psa pathogenicity or the mechanisms of susceptibility/tolerance of kiwifruit plants to Psa. For example, recent studies have contributed to the understanding on how Psa utilizes signal receptors for host recognition, quorum sensing and induction of virulence responses through sensing plant- and bacterial-derived molecules, which inclusively allow Psa cells to communicate with other bacteria (Fiorentini et al., 2019; Cellini et al., 2020; Cellini et al., 2022). Hence, interfering with these bacterial communication systems could result in diminishing the KBC incidence.

In sum, all the strategies being developed for controlling Psa have inherent strengths, weaknesses, opportunities and threats (SWOT) which need to be considered in designing effective and sustainable KBC management (**Table 1**), ultimately providing the necessary tools to growers and phytosanitary surveillance programs.

**Table 1:** SWOT analysis of different plant disease management tools currently being used or under development for the mitigation of the kiwifruit bacterial canker, caused by the bacterium *Pseudomonas syringae* pv. *actinidiae* (Psa).

	Strengths	Weaknesses	Opportunities	Threats
Chemical control				
**Antibiotics**	• High antimicrobial activity against Psa • High specificity	• Existing Psa resistance	• Novel formulations with higher efficacy against Psa	• Environmental impact • Human health concerns • Use restriction
**Copper compounds**	• High antimicrobial activity against Psa • Cost effectiveness • Chemical stability of the formulations	• Existing Psa resistance • Non long-lasting protection under rainy conditions • Risk of phytotoxicity	• Novel formulations with higher efficacy against Psa	• Environmental impact • Human health concerns
Sustainable disease management tools				
**Early disease detection tools**	• Non-invasive • Anticipated and more efficient action • Chemical-free	**•** High cost • Need for high technical knowledge • Variable effectiveness during crop cycle • Data correction and modelling often required	• Poorly explored in *Actinidia*-Psa **•** Technological development • Improvement with machine learning • Combination with unmanned vehicles • Availability of several techniques	• Emergence of strains with different spectral signatures
**Forecast models for Psa risk assessment**	• Non-invasive • Anticipated and more efficient action • Chemical-free	• Low resolution • Need for high technical knowledge • Need for calibration and validation	• Combination with sensing systems • Availability of databases	**•** Unpredictability of climate change
**Plant breeding – conventional approaches**	• Long-term strategy • Chemical-free	• Laborious process	• Genetic wealth of *Actinidia* germplasm	• Risk of losing relevant cultivar traits • Lack of suitable breeding lines
**Plant breeding – gene-editing techniques**	• Long-term strategy • Precise genetic modifications • Faster processes than conventional breeding	• Poor understanding of resistance mechanisms • Need for high technical knowledge • Resistance likely a polygenic trait	• Poorly explored in *Actinidia*-Psa • Development of existing techniques and emergence of novel ones • Availability of information technology	• Restriction of genetically-modified organisms • Low public acceptance
**Plant elicitors**	• Promote early plant defense response • Can be from natural origin • Can be polyfunctional	• Low or no bactericidal effect against Psa • Uncertain stability of the formulations	• Poorly explored in *Actinidia*-Psa	• Risk of phytotoxicity • Costs may be high
**Essential oils and other bioactive compounds**	• High abundance of substances • Natural origin • Can act synergistically	• Variability of their composition • Uncertain stability of the formulations • Poor curative action	• Improvement of EO products through nanotechnology • High abundance of bioactive molecules in their composition	• Risk of phytotoxicity
**Antimicrobial peptides**	• Can be polyfunctional • Promote early plant defense response	• Uncertain stability of the formulations	• High abundance of peptides • Poorly in *Actinidia*-Psa	• To date, none was proven effective against Psa • Risk of phytotoxicity
**Microbial biological control agents – phages**	• High abundance of microorganisms • Chemical-free	• Uncertain stability of the formulations • Broad host range activity	• Screening for novel microorganisms and combinations	• Incorporation of genetic information into the pathogen´s genome • Legislation • Difficulties in upscaling
**Microbial biological control agents – fungi and bacteria**	• High abundance of microorganisms • Chemical-free	• May be affected by other treatments	• Screening for novel microorganisms and combinations	• Risk of ecological imbalances • Legislation • Difficulties in upscaling

## 4 Conclusion

Plant breeding is a cornerstone approach for achieving the milestone of reducing the chemical pesticides application (in 50% by 2030) to meet the European Green Deal. Indeed, breeding for tolerant genotypes (whenever possible) leads to lesser use of phytosanitary products in disease management routines, protecting the environment and human-health. In the case of KBC, the recent gradual introduction of tolerant cultivars had a major contribution in controlling Psa dissemination reducing the threat of this disease. Despite the controversies surrounding gene-editing techniques, these are contributing to further understand fundamental aspects of the plant-pathogen interactions.

Also aligned with the European Green Deal, the search for novel substances to manage KBC is being mostly focused on molecules of natural origin. In this respect, plant elicitors, EOs and other bioactive compounds, AMPs and MBCAs (phages, fungi and bacteria) have shown potential to be used against Psa, and the complexity of their matrices and the enormous possibilities of combination of active ingredients represent a very appealing basis of work for researchers dealing with the *Actinidia*-Psa pathosystem. Indeed, the plant protection industry is currently undergoing a profound transformation and MBCAs and their metabolites will certainly be among the main active ingredients of next-generation PPP to combat Psa.

Emergent technologies such as nanotechnology, for the development of innovative formulations, and artificial intelligence, to be used in the analysis of large amounts of data, will certainly be subject of further development in the next years, and be included in control strategies against Psa in commercial orchards. Increasingly sophisticated disease surveillance tools based on monitoring plant optical properties and forecast models have been gradually implemented in KBC management, and most likely will become a staple in coming years. They hold promise to assist in reducing production losses and containing pathogen spread by means of anticipated sanitary action in the field. Notwithstanding, these novel trends in KBC control are currently at different stages in terms of development, demonstration and deployment. Moreover, the pathogen-host-environment interplay has become increasingly dynamic and unpredictable in face of climate change and food trade globalization. Thus, the plant morpho-physiological, biochemical and molecular mechanisms underlying Psa tolerance must be thoroughly understood to allow developing optimized management regimens, combining different methodologies to tackle distinct risk factors, rather than relying upon one or two approaches alone. Finally, the potential threat of Psa evolving to more virulent biovars, or resistant lineages to existing control methods, highlights the importance of keeping on exploring effective and more environmentally friendly tools for KBC management. We believe that this review will help researchers in better understanding the mode of action, opportunities and limitations of different biotechnological and precision agriculture tools. Moreover, it supports the identification of knowledge gaps and avenues to propel further scientific and technological advances for managing KBC and preventing future outbreaks.

## Author contributions

MS: Data curation, Formal analysis, Validation, Investigation, Writing – original draft. MN: Data curation, Formal analysis, Investigation, Validation, Writing – original draft. MV: Supervision, Validation, Writing – review & editing. SC: Conceptualization, Funding acquisition, Resources, Supervision, Validation, Writing – review & editing.
