# Supplementary material for: Scientific and technological advances in the development of sustainable disease management tools: a case study on kiwifruit bacterial canker
Source: Front Plant Sci. 2024 Jan 11;14:1306420. doi: 10.3389/fpls.2023.1306420 (PMC10808555; doi:10.3389/fpls.2023.1306420)
Supplement: Supplementary file 1 [file Table_1.docx]

Supplementary Information

Table S1: Overview of different plant elicitors, molecules and Microbial Biological Control Agents (MBCAs) that have been tested in laboratory (L), protected cultivation (PC) and field (F) trials to control *Pseudomonas syringae* pv. *actinidiae* (Psa), as alternatives to copper-based products and antibiotics.

| **Elicitors, molecules and MBCAs** | **Mode of application** | **Mode of action** | **Observations** | **References** |
| --- | --- | --- | --- | --- |
| **Plant elicitors** | |  | | |
| Acibenzolar-S-methyl (ASM) | ***In vitro*:** leaf disks test; ***In vivo*:** growth chamber trial (plantlet immersion); greenhouse trial (foliar spraying; root application); field trial (foliar spraying) | Elicitation of the SA-pathway | **L:** no antibacterial activity observed in leaf disks assay; **PC:** decreased symptoms and incidence; decreased endophytic population of Psa; decreased migration of Psa within plant tissues; up-regulation of genes involved in defence mechanisms; best protection provided by root application; callose deposition; superoxide burst; genotype-dependent response; phytotoxicity and promotion of disease development observed; decreased plant growth; **F:** decreased symptoms | Cellini *et al*., 2014; KVH, 2013; KVH, 2015; KVH, 2017; KVH, 2018; Monchiero *et al*., 2015; Collina *et al*., 2016; Wurms *et al*., 2017; Michelotti *et al*., 2018; De Jong *et al*., 2019; Brunetti *et al*., 2020 |
| ASM + *Aureobasidium pullulans* | ***In vivo*:** greenhouse trial (foliar spraying and root application) | Plant elicitation; competition against Psa | **PC:** decreased symptoms with synergistic effect between both active ingredients; decreased Psa epiphytic and endophytic population; decreased plant growth | De Jong *et al*., 2019 |
| ASM + copper compounds | ***In vivo*:** field trial (foliar spraying) | Elicitation of SA-pathway; antibacterial activity | **F:** decreased symptoms; phytotoxicity observed | Monchiero *et al*., 2015; KVH, 2018 |
| B-aminobutyric acid (BABA) | ***In vitro*:** plate assays; leaf disks test | Elicitation of the SA-pathway | **L:** no antibacterial activity observed in plate assay; decreased and delayed Psa symptoms in leaf disks assay | Brunetti *et al*., 2020 |
| Chitosan and chitosan-based products | ***In vitro*:** micropropagated plants (chitosan-enriched medium);  ***In vivo*:** greenhouse trials (foliar spraying; soil amendments); field trial (foliar spraying) | Elicitation of systemic acquired resistance | **L:** overexpression of genes involved in defence mechanisms; **PC:** decreased symptoms; overexpression of genes involved in defence mechanisms; **F:** decreased symptoms; no phytotoxicity observed | Scortichini, 2014; Collina *et al*., 2016; Beatrice *et al*., 2017 |
| Ethylene (ET) | ***In vivo*:** greenhouse trial (fumigation) | Plant elicitation | **PC:** increased symptoms | Cellini *et al*., 2014 |
| Harpin protein + silver | ***In vivo*:** field trial | Plant elicitation | **F:** no effect on disease development. | KVH, 2015 |
| Methyl-jasmonate (MJA) | ***In vitro*:** plate assays; leaf disks test; micropropagated plants (MJA-enriched medium);  ***In vivo*:** greenhouse trial (fumigation) | Elicitation of JA-pathway; weak antibacterial activity | **L:** *in vitro* weak control of Psa growth; increased symptoms in leaf disk assay; overexpression of genes involved in defence mechanisms; **PC:** increased symptoms; genotype-dependent response; overexpression of genes involved in defence mechanisms | Cellini *et al*., 2014; Beatrice *et al*., 2017;  Brunetti *et al*., 2020 |
| Saccharin | ***In vitro*:** plate assays; leaf disks test | Plant elicitation; antibacterial activity | **L:** *in vitro* weak control of Psa growth; decreased and delayed Psa symptoms in leaf disk assay | Brunetti *et al*., 2020 |
| Salicylic acid (SA) | ***In vitro*:** plate assays; leaf disks test; micropropagated plants (SA-enriched medium); ***in vivo*:** greenhouse trial (foliar spraying; root application) | Elicitation of SA-pathway; antibacterial activity | **L:** *in vitro* control of Psa growth; no antibacterial effect in leaf disks test; overexpression of genes involved in defence mechanisms; **PC:** decreased symptoms; overexpression of genes involved in defence mechanisms; genotype-dependent response; no phytotoxicity observed; best protection provided by foliar spraying compared with root application | Cellini *et al*., 2014; Beatrice *et al*., 2017; Brunetti *et al*., 2020 |
| SA + benzalkonium ammonium chloride + urea + propiconazole | ***In vivo*:** field trial (foliar spraying) | Elicitation of SA-pathway; antibacterial activity | **F:** decreased symptoms | KVH, 2013 |
| 1-aminocyclopropane-1-carboxylic acid (ACC) | ***In vitro*:** micropropagated plants (ACC-enriched medium) | ET precursor; plant elicitation | **L:** overexpression of genes involved in defence mechanisms | Beatrice *et al*., 2017 |
| **Essential oils and other bioactive compounds** | | | | |
| *Acorus gramineus; Ammi visnaga; Amomum kravanh; Artemisia capillaris; Asarum sieboldii; Cacalia ainsliaeflora; Carum carvi; Chamaecyparis obtuse; Chamaecyparis pisifera; Chenopodium ambrosioides; Eucalyptus citriodora; Eucalyptus dives; Eucalyptus globulus; Eucalyptus radiate; Eucalyptus smithii; Juniperus chinensis; Juniperus chinensis var. globosa; Juniperus chinensis var. kaizuka; Juniperus chinensis var. sargentii; Juniperus rigida; Kaempferia galangal; Kunzea ericoides; Leptospermum petersonii; Leptospermum scoparium; Melaleuca dissitiflora; Melaleuca quinquenervia; Melaleuca uncinata; Myristica fragrans; Myrtle communis; Paeonia suffruticosa; Pinus densiflora; Pinus koraiensis* | ***In vitro*:** plate assays | - | **L:** no antibacterial activity against Psa | Song *et al*., 2016 |
| *Allium sativum; Cinnamomum* *zeylanicum*; *Foeniculum vulgare*; *Mentha* *spicata*; *Origanum vulgare*; *Ocimum* *basilicum*; *Syzygium aromaticum*; *Thymus vulgaris* | ***In vitro*:** plate assays | Antibacterial activity | **L:** control of Psa growth | Pucci *et al*., 2018 |
| *Anethum graveolens; Coriandrum sativum*; *Cuminum cyminum*; *Eucalyptus polybractea* | ***In vitro*:** plate assays | Antibacterial activity | **L:** control of Psa growth | Pucci *et al*., 2018 |
| *Apium graveolens, Boswellia carterii, Cinnamomum* *camphora*, *Citrus limon, Cymbopogon nardus, Elettaria cardamomum, Lavandula angustifolia, Levisticum officinale, Liquidambar* spp., *Mentha piperita, Origanum majorana, Piper nigrum, Pogostemon patchouli, Salvia officinalis, Santalum album, Zingiber officinale* | ***In vitro*:** plate assays | - | **L:** no antibacterial activity against Psa | Pucci *et al*., 2018 |
| *Camellia sinensis* extract | ***In vitro*:** plate assays; ***In vivo*:** growth chamber (foliar spraying) | Antibacterial activity | **L:** control of Psa growth; reduction of Psa siderophore activity and lipase activity; reduction of Psa motility and biofilm formation; downregulation of Psa virulence genes; *In vivo* (growth chamber): decreased symptoms and disease incidence | Lovato *et al*., 2019 |
| Chebulagic acid, chebulinic acid; corilagin; methyl gallate; quercetin 3-O-α-L-arabinopyranoside; tercatain | ***In vitro*:** plate assays | Bactericidal | **L:** control of Psa growth | Vu *et al*., 2017 |
| Cinnamaldehyde | ***In vitro*:** plate assays;  ***In vivo*:** greenhouse trial (foliar spraying) | Bactericidal | **L:** control of Psa growth  **PC:** decreased symptoms | Collina *et al*., 2016; Song *et al*., 2016 |
| *Cinnamomum cassia*; *Melaleuca cajuputii*; *Melaleuca linariifolia*; *Pimenta* *dioica*, *P. racemosa* | ***In vitro*:** plate assays | Antibacterial activity | **L:** control of Psa growth | Song *et al*., 2016 |
| Citrus extracts + palm oil | ***In vivo*:** field trial (foliar spraying) | - | **F:** no effect on visual symptoms | KVH, 2013 |
| *Curcuma longa*; *Ginkgo biloba*; *Gynostemma pentaphyllum*; *Paullinia cupana*; *Theobroma cacao* (extracts) | ***In vitro*:** leaf disks test | - | **L:** no effect in leaf disk assay in all substances | Brunetti *et al*., 2020 |
| Ellagic acid | ***In vitro*:** plate assays; ***In vivo*:** greenhouse trial (foliar spraying) | Antibacterial activity | **L:** control of Psa growth; **PC:** decreased symptoms and Psa epiphytic population; improved efficacy with microencapsulation | Rossetti *et al*., 2017 |
| Estragole, eugenol, methyl eugenol | ***In vitro*:** plate assays | Bactericidal | **L:** control of Psa growth | Song *et al*., 2016 |
| Extract from plants from the Liliales order (Genera: *Allium*, *Aloe* and *Asparagus*) and extract from plants from the Urticales order (Genera: *Dorstenia*, *Ficus* and *Morus*) | ***In vitro*:** plate assays; ***In vivo*:** greenhouse trial (foliar spraying) | Antibacterial activity | **L:** control of Psa growth; **PC:** control of Psa growth | Balestra, 2007 |
| Forsythoside A (from *Forsythia suspensa*) | ***In vitro*:** plate assays;  ***In vivo*:** growth chamber trial (foliar infiltration) | Bactericidal | **L:** reduction of Psa motility and biofilm formation; decreased transcription of Psa virulence genes; interference in the bacterial metabolism; deformity and rupture of Psa cells;  **PC:** decreased symptoms in leaf plate assay | Pei *et al*., 2022 |
| Gallic acid | ***In vitro*:** plate assays; ***In vivo*:** greenhouse trial (foliar spraying) | Antibacterial activity | **L:** control of Psa growth; **PC:** decreased symptoms and Psa epiphytic population; improved efficacy with microencapsulation | Vu *et al*., 2017; Rossetti *et al*., 2017 |
| Gallic acid and ellagic acid (mixture) | ***In vitro*:** plate assays;  ***In vivo*:** greenhouse trial (foliar spraying); field trial (foliar spraying) | Antibacterial activity | **L:** control of Psa growth; **PC:** decreased symptoms and Psa epiphytic population; **F:** decreased symptoms and Psa epiphytic population; improved efficacy with microencapsulation | Rossetti *et al*., 2017 |
| *Melaleuca alternifolia* | ***In vitro*:** plate assays | Antibacterial activity | **L:** control of Psa growth | Pucci *et al*., 2018;  Vavala *et al*., 2016 |
| *Mentha* *suavelens* EO; Salvia EO; Lauro EO | ***In vitro*:** plate assays | Antibacterial activity | **L:** control of Psa growth | Vavala *et al*., 2016 |
| *Monarda* *didyma* EO; *Monarda fistulosa* EO | ***In vitro*:** plate assays | Antibacterial activity | **L:** control of Psa growth | Mattarelli *et al*., 2017 |
| *Morus nigra* (extract) | ***In vitro*:** plate assays | - | **L:** neither bacteriostatic nor antibactericidal activity | Simonetti *et al*., 2020 |
| Myrcene; 4-allylphenol; β-caryophyllene; limonene; linalool; α-pinene; 6-methyl-5-hepten-2-one; 1.8-cineole; α-phellandrene; ρ-cymene; α-humulene; terpinen-4-ol; α-terpineol; geraniol; copaene | ***In vitro*:** plate assays | - | **L:** no antibacterial activity against Psa | Song *et al*., 2016 |
| *Phyllostachys* *heterocycla* f. *pubescens* (extracts) | ***In vitro*:** plate assays | Antibacterial activity | **L:** control of Psa growth | Mori *et al*., 2019 |
| *Polygonum cuspidatum*; *Vitis vinifera*; *Hypericum perforatum* (extracts) | ***In vitro*:** plate assays; phytotoxicity assay (leaf disks test) | Bactericidal and bacteriostatic | **L:** control of Psa growth; elicitation with chitosan oligosaccharides improved the performance of *H. perforatum* extract; no phytotoxicity except for extract from elicited *H. perforatum* | Simonetti *et al*., 2020 |
| Propolis (unspecified origin) | ***In vivo*:** greenhouse trial (foliar spraying) | Bactericidal | **PC:** decreased symptoms, no signs of phytotoxicity | Monchiero *et al*., 2015 |
| *Punica granatum* (extract) | ***In vitro*:** plate assays | Bacteriostatic | **L:** bacteriostatic effect; no antibactericidal activity observed | Simonetti *et al*., 2020 |
| *Rosmarinus* *officinalis* | ***In vitro*:** plate assays | Antibacterial activity | **L:** control of Psa growth | Vavala *et al*., 2016 |
| *Satureja* sp., *T. vulgaris* (individual assessment) | ***In vivo*:** greenhouse trial (foliar spraying) | Bactericidal | **PC:** decreased symptoms | Monchiero *et al*., 2015 |
| Thymol-based extract | ***In vivo*:** greenhouse trial (foliar spraying) | Bactericidal | **PC:** decreased symptoms | Collina *et al*., 2016 |
| **Antimicrobial peptides (AMPs)** | | | | |
| AMPs | ***In vitro*:** plate assays; leaf disc assay; ***In vivo*:** climate-chamber trial (diffusion through leaf wounds); field trial (foliar spraying) | Antibacterial activity; bactericidal | **L:** control of Psa growth; antibiofilm activity; synergism observed in AMPs combination; disruption of Psa membrane integrity; down-regulation of Psa genes related to bacterial replication and damage recovery; decreased Psa symptoms and Psa load in leaf disc assay;  **PC:** decreased Psa symptoms and Psa load in leaves;  **F:** decreased symptoms (utilized AMPs were not disclosed) | Cameron *et al*., 2014; Zoysa *et al.*, 2015; KVH, 2017; Camó *et al*., 2018; Oliveras et al., 2018; Mariz-Ponte *et al*., 2021 ; Zhang *et al*., 2023a |
| **MBCAs – phages** | |  |  |  |
| Families of phages: Cystoviridae, Podoviridae, Siphoviridae, Myoviridae | ***In vitro*:** plate assays; viability assessment (pH, temperature; radiation); leaf discs assays;  ***In vivo*:** greenhouse trial (foliar spraying) | Bacteriophagy | **L:** control of Psa growth; bacterial load reduction and decreased Psa symptoms in leaf tissues *in vitro*; **PC:** bacterial load reduction and decreased Psa symptoms in leaves | Frampton *et al*., 2014, Di Lallo *et al*., 2014; Yu *et al*., 2016; Park *et al*., 2018; Yin *et al*., 2019; Pinheiro *et al*., 2019; Flores *et al*., 2020; Song *et al*., 2021; Fiorillo *et al*., 2023 |
| **MBCAs – fungi and bacteria** | | | | |
| *Aureobasidium pullulans* | ***In vitro:*** plate assays; ***In vivo*:** greenhouse and field trials (foliar spraying) | Plant elicitation; competition against Psa | **L:** control of Psa growth; **PC:** decreased symptoms; overexpression of genes involved in defence mechanisms; decreased epiphytic population of Psa; **F:** decreased symptoms | Collina *et al*., 2016; Hoyte *et al*., 2018; De Jong *et al*., 2019 |
| *Bacillus amyloliquefaciens; B. subtilis.* | *In vivo*: greenhouse trial and field trial (foliar spraying) | Competition against Psa; plant elicitation | **PC:** decreased symptoms | Monchiero *et al*., 2015; Collina *et al*., 2016 |
| *Glomus* spp. + *B. amyloliquefaciens +* *Streptomyces* spp. + *Trichoderma harzianum* | ***In vivo*:** greenhouse trial (foliar spraying) | Competition against Psa; plant elicitation | **PC:** decreased symptoms | Collina *et al*., 2016 |
| *Glomus* spp*.* + *B. subtilis* + *Streptomyces* spp. + *Pseudomonas* spp. + *Trichoderma* spp. + *Pichia pastoris* | ***In vivo*:** greenhouse trial (foliar spraying) | Competition against Psa; plant elicitation | **PC:** decreased symptoms | Collina *et al*., 2016 |
| *Pseudomonas putida*; *Ulocladium oudemansii* | ***In vivo*:** field trial (foliar spraying) | - | **F:** no effect on visual symptoms | KVH, 2013 |
| *Trichoderma* spp. | ***In vivo*:** growth chamber trial and field trial (application at the stem base) | Plant elicitation; competition against Psa | **PC:** decreased symptoms and increased survival; genotype-dependent response; **F:** decreased symptoms; synergistic effect with plant elicitors and other beneficial microorganisms (unspecified) | Hill *et al*., 2015 |
| **Other molecules/compounds** | | | | |
| 1-methylcyclopropene (1-MCP) | ***In vivo*:** greenhouse trial (fumigation) | Inhibitor of ET perception | **PC:** decreased symptoms | Cellini *et al*., 2014 |
| Abscisic acid (ABA) | ***In vivo*:** greenhouse trial (foliar spraying; root application) | - | **PC:** increased symptoms with foliar spraying; decreased symptoms with root application. | Cellini *et al*., 2014 |
| Forchlorfenuron + copper hydroxide | ***In vivo*:** field trial (foliar spraying) | Plant growth regulator + antibacterial compound | **F:** decreased symptoms | KVH, 2018 |
| Gentisic acid | ***In vitro*:** plate assays; leaf disks test | - | **L:** *in vitro* weak control of Psa growth | Brunetti *et al*., 2020 |
| Indole-3-acetic acid (IAA) and IAA-like molecules (triclopyr) | ***In vitro*:** plate assays; leaf disks test | Plant growth regulators | **L:** *in vitro* control of Psa growth with IAA; no effect of IAA in leaf disk assay; increased symptoms in leaf disk assay with triclopyr | Brunetti *et al*., 2020 |
| Other plant hormones, namely 1-Naphthaleneacetic acid (NAA), gibberellic acid (GAA), 1-aminocyclopropane-1-carboxilic acid (ACC), jasmonic acid (JA) and kinetin (cytokinin) | ***In vitro*:** plate assays; leaf disks test | Plant growth regulators | **L:** *in vitro* weak control of Psa growth with NAA, GAA and ACC; no effect on Psa growth *in vitro* with JA and kinetin; no effect in leaf disk assay in all substances | Brunetti *et al*., 2020 |
| Phytoalexins | ***In vivo*:** field trial (foliar spraying) | Antimicrobial | **F:** decreased symptoms (discrete effect) | KVH, 2015 |
| Polyamines (cadaverine, L-Ornithine, putrescine, spermidine, spermine) | ***In vitro*:** plate assays; leaf disks test | Cellular processes regulator | **L:** *in vitro* control of Psa growth with cadaverine and spermidine; no effect of putrescine and spermine on Psa growth; *in vitro* weak control of Psa growth with L-Ornithine | Brunetti *et al*., 2020 |
| Prohexadione-Ca | ***In vivo*:** greenhouse trial (foliar spraying) | Plant growth regulator | **PC:** no effect on visual symptoms | Collina *et al*., 2016 |
| Salicylate derivatives | ***In vivo*:** field trial (foliar spraying) | - | **F:** no effect on Psa growth | KVH, 2013 |
| Silicon-based compounds (silica, talcum powder and pumice) | ***In vitro*:** plate assays; leaf disks test | - | **L:** no effect on Psa growth | Brunetti *et al*., 2020 |
| Zinc-copper-hydracid of citric acid biocomplex | ***In vivo*:** field trial (foliar spraying) | - | **F:** decreased symptoms | Scortichini, 2016 |

References

Kiwifruit Vine Health (2013). 2012/13 potted plant field trial report (Mount Q19 Maunganui, New Zealand: KVH and Zespri Group Ltd).

Kiwifruit Vine Health (2015). 2014/2015 potted plant field trial report: elicitors on Gold3 potted plants (Mount Maunganui, New Zealand: KVH and Zespri Group Ltd).

Kiwifruit Vine Health (2017). 2015/16 potted plant field trial report. Actigard and Estim rates on Bruno and Gold3 potted plants (Mount Maunganui, New Zealand: KVH and Zespri Group Ltd).

Kiwifruit Vine Health (2018). 2018 potted plant field trial report. Efficacy of tank mixes on Psa control in Hayward and Gold3 potted plants (Mount Maunganui, New Zealand: KVH and Zespri Group Ltd).
